# Laypeople's Collaborative Immersive Virtual Reality Design Discourse in Neighborhood Design

**DOI:** 10.3389/frobt.2019.00097

**Published:** 2019-10-14

**Authors:** Shuva Chowdhury, Marc Aurel Schnabel

**Affiliations:** School of Architecture, Victoria University of Wellington, Wellington, New Zealand

**Keywords:** immersive virtual environment, design participation, communication, laypeople, protocol analysis, urban design

## Abstract

The article discusses design communication and participation of laypeople in a virtual participatory urban design process. We speculate that an immersive virtual environment facilitated instrument can allow laypeople to take part actively as designers in the early stage of urban design ideation and generation. We have developed a design communication framework where laypeople can participate in design discourse on a neighborhood's future urban form. The strategy describes an urban design intent, which is informed by the development procedure of an instrument and workflow to engage participants. The integration of the instrument and the engagement procedure enable continuous designing of urban form by laypeople. A protocol analysis has been undertaken to investigate design communication. A coding scheme is applied to investigate, analyse, and understand how laypeople communicate with the design instrument and control design in the virtual environment. Through engaging non-experts, the research impacts on the perceptual affordance created by immersive 3D buildings artifacts and verbal conversation. The protocol analysis validated the setup so that subsequent studies can address the meaningfulness of such design conversations.

## Introduction

Participatory design techniques deal with urban issues often used paper-based methods (Al-Kodmany, 2001) and depended on digitally produced images or three-dimensional artifacts (Bannon et al., 2018). The demand for public participation in the urban design decision-making process brings accountability on the parts of stakeholders (Healey, 1998; Murray et al., 2009). However, the lack of visual information and tools in the design process prevents the end-users from taking part in design as they inhabited the environment. Furthermore, conventional urban design processes do not allow for laypeople to take part in the design ideation and generation stage. So, we speculate that an Immersive Virtual Environment (IVE) facilitated instrument enables laypeople to actively take part as designers in the early stage of urban design process. The research has been framed to accommodate an urban design task in accordance with the designing scope of the IVE instrument. Nevertheless, we recognize that it is also impossible for urban designers to address all aspects of urban dynamics in a single design process (Chowdhury and Schnabel, 2018).

The study develops a design discussion platform for non-experts to produce urban forms by employing virtual tools. Quality urban design depends significantly on social, economic, and environmental issues. Traditional urban design tools are not flexible enough to address design changes in the early design stages and have spatial and temporal limits in their capacity to share design ideas. Moreover, they do not allow end-users to participate in the early design iteration stages. Our research engages laypeople to take part in the design imagination and generation of a neighborhood in IVE. These tools offer a dynamic virtual interactive platform by which to visualize and produce iterative design ideas. We discovered that engaging community members in this way enabled them to easily work together to create different designs, and to collaborate naturally, including on important perhaps less exciting design elements like driveways and fences.

As a case study, we considered the suburb of Karori in Wellington, New Zealand. In Karori, Wellington City Council (WCC) has run year-long charrettes to better understand community interests and priorities and to identify locations for further development (Karori, 2017; Wellington City Council, 2017). To date, the charrette process has generated a map of priorities within the Karori neighborhood, and the mall area has been signaled as a priority for redevelopment (Karori, 2017). Our research includes an empty lot in Karori centre as the context for new design ideas in the IVE participatory platform.

## Collaborative Immersive Virtual Environments

Immersive Virtual Environment gives the experience of sensed reality in virtual environments. It helps the user to perceive some volumetric qualities of a building or space which are hard to depict in 2D drawings. It develops an artificial environment that imitates real-world surroundings convincingly enough that the users suspend skepticism and fully engage with the created environment. IVE offers an active and real-time interaction with the design, therefore presenting an authentic feeling of being in the environment. It has proved that the qualities of design and the designed products are directly linked to the nature of the communication and collaboration which has taken place during the design process (Schnabel and Kvan, 2003). IVE's three-dimensional (3D) medium leverages users to create, communicate and collaborate during the design process. It already has shown significant contribution in the field of architectural practices for design communication with stakeholders. Design communication during the design process plays a substantial role in the exchange of messages and ideas between people with different skillsets and interests. Using visualization during this process provides an effective way to communicate information, thus generating more creative ideas.

Perceptual awareness is an important factor in IVE design collaboration, as evidenced in ethnographic studies (Maher, 2011). This is because independent participants in the collaborative design process need to be able to coordinate and inform their activities through background or peripheral awareness of one another's activities. So, Collaborative Virtual Environments (CVE) provide new ways to meet communication needs when negotiation is important and frequent. An important aspect of collaborative design is that the focus of the meeting is on the design ideas and models rather than only discussion between designers. It is necessary to develop a shared understanding of the design problem and potential solutions. However, communication among the participants in the environment allows individuals to pursue their own tasks as well as to focus their attention on a shared task. Also, studies report that designers move fluidly from working individually to working together when engaged in virtual collaborative design.

There is a significant cognitive impact with regards to collaborative virtual environments. Research done by Gül and Maher (2009) shows that analysis of the collaborative design protocols provides a basis for better understanding the interactions with different representation techniques. The acquired knowledge has implications for both developments in future collaborative virtual environments and for choosing an appropriate medium for designing.

Hemmerling (2018) argues that regardless of the content, new technologies cause changes in perceptions and thinking. He refers to McLuhan's quote, “we shape our tools and then our tools shape us.” The development of communication media tools acts as a controlling force for social change. The content in the digital media influences the spaces in which we live, the surrounding objects, the images and the sounds. This means that users acquire different understandings of the content due to the differences in the tools. So, designing communication tools for users requires new ways of thinking, feeling, and working.

## Research Methodology

Within a framework of qualitative research, a series of surveys and experiments were set up to investigate the scope of laypeople's active design ideation, generation and collaboration in neighborhood design. The methodology incorporates a survey of urban design consultation, developing the VR instrument, engaging laypeople in IVE urban design, a survey on IVE engagement, an audio recording of the design conversation, transcribing recorded data, protocol analysis, and expert evaluation. This article reports on the development and engagement of laypeople in IVE neighborhood design and the outcome of the protocol analysis of the IVE design communication and participation.

### Design Instrument

Due to its flexibility to create iterative 3D models through hand gesture, an immersive instrument has been developed using in-game-engine software “Unity3D.” The initial modeling technique has been described by Innes et al. (2017). We have adapted parts of it and extended it to the surrounding urban context. One person at a time is immersed in the virtual environment, whilst the other person visualizes real-time design output on a 2D display screen and provides verbal feedback to the first participant. The conversation in the design sessions is audio recorded. The method is akin to Schnabel's (2011) immersive virtual environment design studio research.

The relevant structures of the Karori Centre were modeled in fine detail to resemble the buildings and the contextual urban elements in the virtual environment. To achieve the expected accuracy of the 3D building models with surrounding information, the geographical information system (GIS) generated a topography which was imported to develop the 3D mesh of the terrain and to position the models on the terrain. The IVE interface is scripted in “Unity3D,” where the participant can select different geometrical shapes to build their objects. The surrounding 3D model information provides continuous feedback to inform subsequent design moves. The participant is able to jump from place to place, look around in the environment and make a decision on building forms by experimenting with geometrical attributes offered by the interface. The interface facilitates the creation of any shape of cuboids and the size is depending on the extent of the participant's reach.

### Experimental Procedure, Task, and Participant

**Figure 1:**
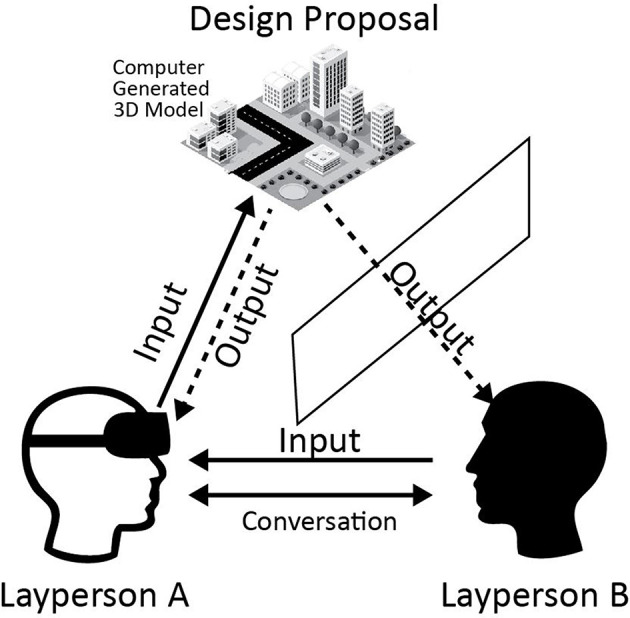
Laypeople design team.

Simultaneously, the immersive design output is projected onto a display screen, which facilitates design collaboration and communication between the participants. The first participant designs by being immersed in the environment via a Head-Mounted Display (HMD). The second participant visualizes the design in real-time on the 80-inch display screen (Figure 1). We recruited participants through social media and poster invitations. Participants were based in New Zealand, of mixed ages, and were familiar with the Karori context.

**Figure 2:**
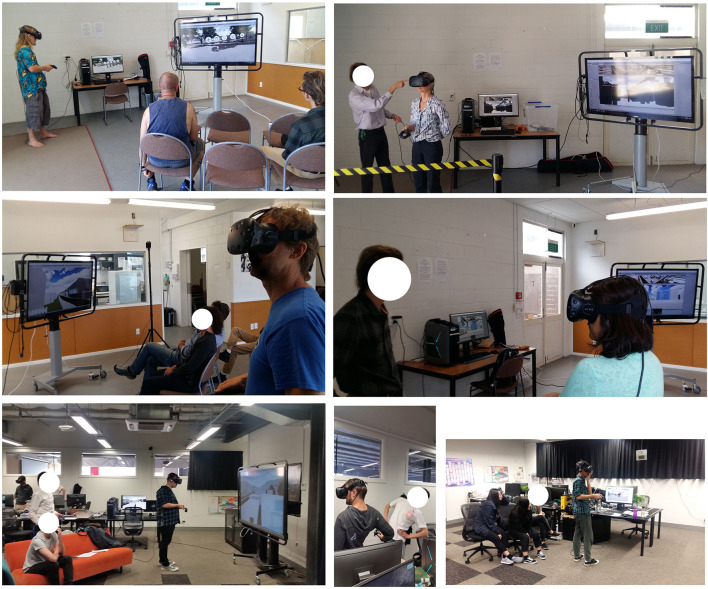
IVE design engagement in Karori Community Centre.

IVE design participation happened in groups (Figure 2). A design task was introduced supporting the contextual requirements. For the Karori suburb, participants were asked to design building blocks on the empty corner plot of Karori Centre. The session began by introducing the participants to the IVE instruments and familiarizing them with the Karori Centre site on a Google map. The participants were allowed to extend their design ideas beyond the assigned plot if they wished. We held IVE design engagement events in the Karori Community Centre to engage local people in the instrument. We recorded the design conversations for protocol analysis. The 3D models produced were saved for experts to evaluate the outcomes.

### Design Coding Process

The coding scheme is based on a set of assumptions about the general structure of the problem-solving processes and the verbal reporting process. In the context of the research, the transcribed conversation has been coded by two people according to the coding scheme and discussions have taken place in cases where discrepancies arose. The research analyses conversations from three sessions for protocol analysis. Derived from Tsai et al. (2009) and inspired by Ericsson and Simon (1993), we have developed a coding scheme to analyse design communication and collaboration based on the conversation recorded during the engagement session in VR instrument. Tsai et al. (2009) have referred to Levinson's (2001) works on defining coding for semantic conversation and handing over the conversation. For example, a speaker may hand over the conversation to another member by asking questions, such as “isn't it?” or statements as “you know,” or by specifically naming the next speaker.

The transcribed conversation was coded in order to organize the data. The coding scheme is applied to investigate, analyse, and understand how laypeople as designers communicate with the design instrument and control design ideas in VE. The four major categories of the scheme are Communication Control, Design Communication, Social Communication, and Communication Technology. The result of the IVE engagement demonstrates the potentiality of the instruments for design communication, whilst presenting the limitation of the communication due to technological discrepancy. The coding scheme is explained below in Table 1.

**Table 1:** The coding scheme for VR collaboration (after Gabriel and Maher, 2002; Tsai et al., 2009).

**Communication control**	**Code**	**Description**
Interruption by design	INT	When a design member interrupts another member.
Interruption by instrument	INTS	When a design member interrupts by instrument functioning. E.g., wrong button/unexpected VR movement/instrument shut down.
Handing-over the conversation	HAN	Handing over the conversation from a design member to another member. Possibly through questions or by specifically naming the next speaker.
Pause	PAU	Pausing during the communication.
Design communication	Code	Description.
**Design concept**		*What is communicated*
Introduction of idea	IDE	When a design member directly or indirectly introduces an idea.
Acceptance of idea	ACC	When a design member accepts an idea of another member.
Rejection of idea	REJ	When a design member does not accept an idea of another member.
Clarification of idea	CLA	When a design member explains why the idea is appropriate.
Seek clarification of idea	CLAS	When a design member seeks clarification of another member's decision.
Development of idea	DEV	When a design member further develops an idea.
Evaluation of idea	EVA	When a design member spends time evaluating an idea.
**Design detail**		*How the concept is created*
Discussion of size	VSZ	When design members discuss the size of the 3D object/building.
Discussion of shape	VSP	When design members discuss the shape of a 3D object/building.
Discussion of movement	VSM	When design members move in the VR environment.
Discussion of type	VST	When design members discuss building types.
Discussion of space	VSS	When design members discuss spatial attributes. E.g., site entry, openness or closeness, orientation, etc.
Discussion of color/texture	VCL/VTXT	When design members discuss the color and texture on a 3D building or parts of it.
**Design task**		*How the design is implemented*
Task questioning	TKQ	When design members ask questions about their design task.
Agenda referring	AAR	When design members refer to the agenda.
Instructing	INS	When a design member instructs another member
Working status	VWS	When design members state what they are currently doing or what they have done. E.g., “I just finished the walls.”
Social communication	Code	Description.
Non-task-related social communication	NRT	When design members talk about non-task-related things.
Joking	JOK	When a design member laughs or makes a joke.
Communication technology	Code	Description.
VR instrument	VTL	When design members discuss the use of tools for design in the VR environment.
Examining	EXA	When a design member examines what has been done by using the instrument.

Communication Control has four subcategories: “Interruption by Design” members (INT); “Interruption by Instrument” (INTS); “Handing-over the Conversation” (HAN); and “Pause” during the communication (PAU). Pause (PAU) is used if there is a temporary cessation of conversation during design collaboration in a virtual environment. We added the section of INTS due to computer and software running interruptions.

The Design Communication scheme has been sub-categorized by Design Concept, Design Details, and Design Tasks. Design Concept includes how design ideas are handled during the design process such as “Introduction of Idea” (IDE), “Acceptance of Idea” (ACC), “Rejection of Idea” (REJ), “Clarification of Idea” (CLA), “Seek Clarification of Idea” (CLAS), “Development of Idea,” and “Evaluation of Idea” (EVA). CLAS was added after Tsai et al. (2009), when the designer asks questions about the design decision to inform the next design move. One recalls that, only one participant is designing at a time as the other participant is providing feedback concurrently.

Design Detail comprises the sub-categories “Discussion of Size” (VSZ), “Discussion of Shape” (VSP), “Discussion of Movement” (VSM), “Discussion of Type” (VST), “Discussion of Space” (VSS) and “Discussion of Color/Texture” (VCL/VTXT). VSZ and VSP have been added to evaluate the perceptual scaler understanding of 3D models produced in the virtual environment. An immersive 1:1 perspectival environment, the understanding of 3D building volumes initiates a different conversation on spatial understanding. Also, the developed interface allows the participant to jump or move in the virtual urban context. The VSM has been added to evaluate that perceptual movement in the virtual environment.

The coding scheme of Design Task includes “Task Questioning” (TKQ), “Agenda Referring” (AAR), “Instructing” (INS), and “Working Status” (VWS). TKQ is used when a design participant asks questions about their design tasks. AAR is used when a design member refers to the design agenda. In this case, the task of designing a mixed-use building block. VWS is used when a design member states what they are currently doing or have done, e.g., “I have just finished the wall.”

Social Communication comprises “Non-task-related Social Communication” (NRT) and “Joking” (JOK) in between conversations. This coding scheme documents the moments of conversation that are not related to design tasks.

The coding scheme of Communication Technology consists of “VR Instrument” (VTL) and “Examining” (EXA). The VTL scheme is used when design participants discuss the use of the instrument. The EXA scheme documents when design participants discuss what they have done using the instrument.

## Analysis of IVE Collaboration

**Figure 3:**
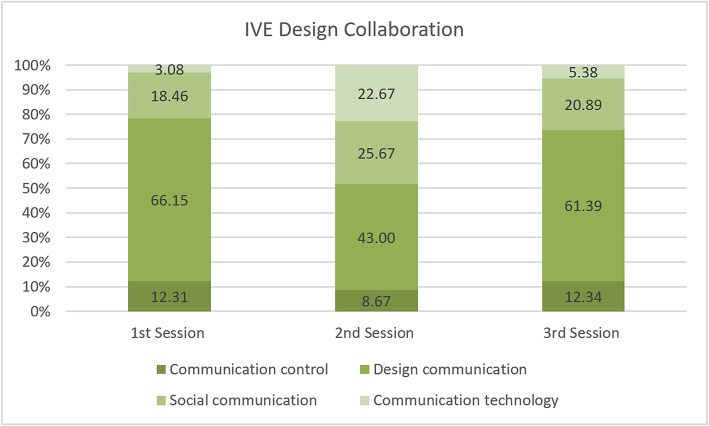
IVE design collaboration.

The coding results from three design sessions show that design communication is a dominant activity in IVE design collaboration. For all three sessions, the highest percentage of coding indicates that design communication happens during the design process (Figure 3). Designers communicated about tasks to design an urban form. They discussed building types, location, height, materials, orientation, etc. The virtual contextual information oriented them to discuss those design tasks. Figure 3 also shows that the conversation happened due to social communication, which is a non-task related discussion between designers. This indicates that the instrument can facilitate the flow of non-relevant discussion such as jokes in an IVE or other non-task related conversation.

Moreover, conversations arose because of difficulties controlling the IVE instruments. The instrument was new for most of the designers. Also, familiarization with the instrument varied from designer to designer which resulted in different percentages for Communication Control coding in the three different sessions. The results also differed in Communication Technology, where the designers had to discuss using the instrument.

**Figure 4:**
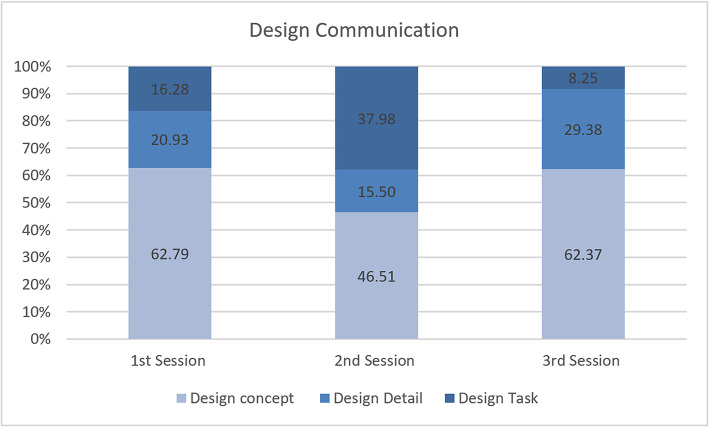
Design communication.

In detail, it seems that most of the design communication happens during design conceptualization (Figure 4). For the three different sessions, the majority of conversation centered on introducing and discussing design concepts. This means that designers directly or indirectly introduced design ideas in immersive virtual space via HMD and sought acceptance on those ideas from other participants via the 80-inch display screen. Designers talked about the introduction, acceptance, rejection, development, explanation, and evaluation of their ideas, often seeking clarification. A substantial percentage of coding shows that the designers discussed design details like the size, shape and material of the 3D object/building, building types, as well as movement in the virtual environment, and the spatial experience. Comparatively, conversations took place that were relevant to the design tasks, where designers asked questions, referred to the task agenda, instructed other designers and stated their working status. The percentage differences for each session indicate that the designers spent more time on developing design concepts compare to the design detail and the design task. This reflects that the instrument can instigate continuous design ideas. However, these differences might also be due to unfamiliarity with the instrument, as designers had to spend most of their time initiating, accepting, rejecting, clarifying, developing, and evaluating design ideas.

**Figure 5:**
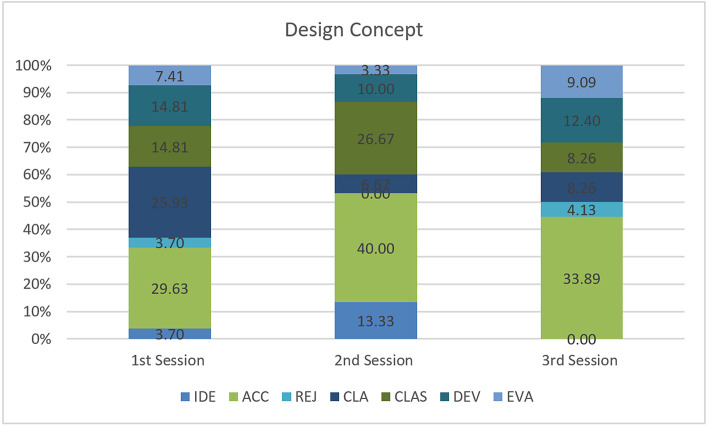
Design concept.

The Design Concept coding results show that the designers spent most of their time accepting design ideas, then evaluating the ideas and introducing ideas. For the three sessions, the highest percentage of communication (during the design concept stage) focused on one member accepting the design idea of another member (Figure 5). This means that when designer A in the IVE asked for consent from designer B who was monitoring design in the 2D display screen, design ideas were successfully communicated. Again, design concept conversations happened when explaining the appropriateness of the design ideas. Similarly, the design concept conversation occurred when seeking clarification regarding another design member's decision. Conversation also took place relating to the development of the design idea. Table 2 shows one of the examples of such a conversation.

**Table 2:** Design conversation regarding pillar and columns.

Designer A:	[JOK]	“…this side, yeah just ‘cause it's easier, eh [laughter]
Designer B:	[CLA]	I think it's because it's the corner of the room and it closes…
Designer A:	[IDE]	Ah, I've got an idea let's make a pillar…
Designer B:	[ACC]	Oh yeah…
Designer A:	[CLA]	…here
Designer B:	[VSP]	…like columns…”

*JOK, Non-task related Joke; CLA, Clarification of Idea; IDE, Introducing Idea; ACC, Acceptance of Idea; VSP, Discussion of Shape*.

**Figure 6:**
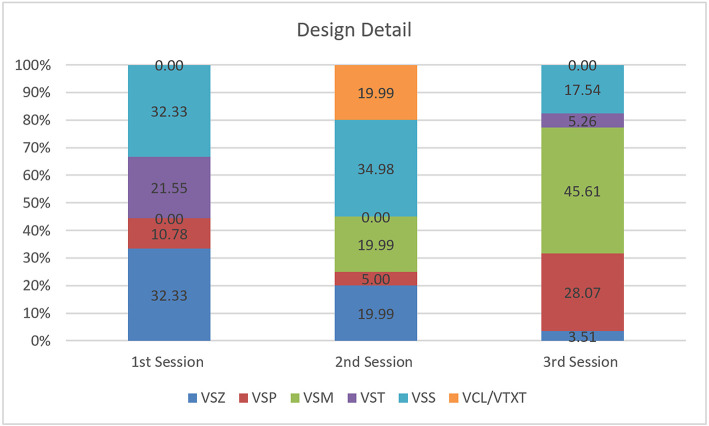
Design detail.

In terms of how the concept is created, a detailed analysis of Design Details confirms that positive communication happened when discussing building shape, spaces, functional type, size, movement, and material texture. Some conversations related to the size of the 3D objects or buildings (Figure 6). Similarly, a conversation about design details centered on the shape of the 3D objects or buildings. These kinds of conversations facilitated the designer's decisions on the types of urban form they were proposing. Table 3 shows one of the examples of such a conversation.

**Table 3:** Design conversation on location and building features.

Designer B:	[08:11]	[VSS]	What would you have under there?
		[VSS]	Um, I guess you could put some café seating…
Designer A:	[08:18]	[DEV]	So it's out of the sun…
Designer B:	[08:21]	[ACC]	Yeah
Designer A:	[08:23]	[IDE]	Because it's Wellington, you probably want to figure out some windbreaks
Designer B:	[08:28]	[DEV]	Yeah, just a giant wall around the entire building
Designer A:	[08:35]	[VSZ]	[laughs] Yeah, just a really big windbreak
Designer B:	[08:43]	[EVA]	But it's Karori though so it's not too windy
Designer A:	[08:44]	[ACC]	Yeah, true.

*EVA, Evaluation of Idea; DEV, Development of Idea; IDE, Introducing Idea; VSZ, Discussion of Size; VSS, Discussion of Space; ACC, Acceptance of Idea*.

In the second and third sessions, the coding data also recorded conversations about movement in the IVE. This means that the designers were able to recognize Karori in the virtual environment, and they could identify the contextual urban elements with the real neighborhood. They were also able to communicate about their movement in that artificial environment. In addition, the result of the coding analysis shows that they spent time trying to understand the space with their proposed alternatives, as conversations arose relating to spatial attributes of the design.

**Figure 7:**
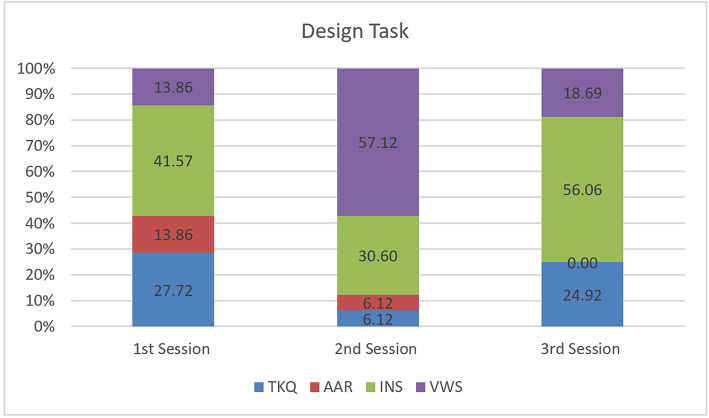
Design task.

Regarding the Design Task, most of the conversation centered on working status. Conversations took place when designers stated what they had done or what they were doing (Figure 7). This shows the presence of design communication as the designers shared their working status. Similarly, the percentages of task-related conversation focused on instruction. Such conversations occurred when a designer gave any design instruction to the other designer. Furthermore, some conversations arose from questions relating to the task.

**Figure 8:**
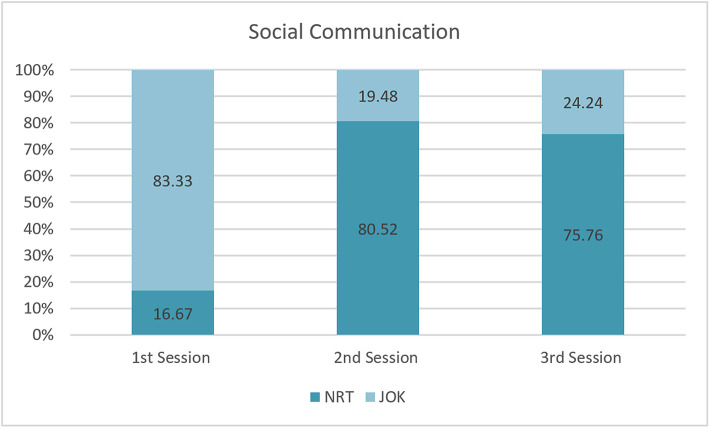
Social communication.

Figure 8 shows that the designers spent a significant amount of time on non-task-related social communication and joking during the design sessions in the IVE instrument. It indicates that there was continuity of conversation during the design session which occasionally gave rise to witty banter. For the first, second and third sessions most of the conversations on Social Communication were coded as Joking. This usually happened when a design member laughed or joked and, similarly, when a conversation of social communication was non-task related. This means that the IVE instrument facilitated the flow of conversation during the design sessions.

**Figure 9:**
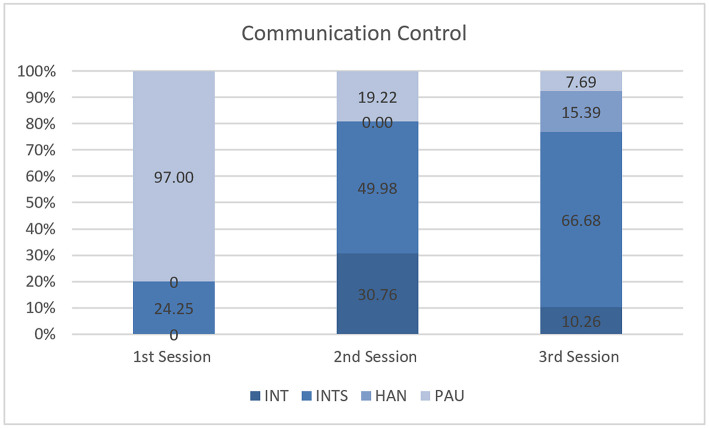
Communication control.

The lack of familiarity with the IVE instrument interrupted the design communication substantially. Design interruption occurred due to pressing the wrong button or from an unexpected VR movement. The highest percentage of conversations happened due to the Interruption by Instrument (Figure 9). Also, the coding indicates that there were incidents of Pause during the design communication.

## Discussion and Conclusion

The significance of this research can be seen from the perspective of communication and producing meaningful design collaboration between laypeople in urban design. Despite only having analyzed three teams, through engaging non-experts, the research evidences that the perceptual affordance of the IVE instrument allowed them to take part and design in the early stage of the urban design process. Through protocol analysis, it has been demonstrated that spontaneous exchange of visual information in a virtually informed environment along with verbal conversation can produce a meaningful design outcome.

### Design Communication

The conversations between the participants were relevant to design intervention, were context related and arose naturally from the synchronized setup of design engagements and task distribution among the participants. The designer acted in the immersive world via HMDs, and fellow participants provided verbal feedback via the 80-inch display screen. In between snippets of conversation, the computer produced 3D urban forms which provided visual feedback to facilitate further discourse. It resulted from the perceptual affordance of the interface supporting Gaver's (1991) concept of “technological affordance,” whereby the 3D artifacts produced possess the perceived properties to influence subsequent design actions. However, it is to be noted that a given percentage of the conversations were disrupted due to technological interruption.

The results also reiterate that verbal communication can perform effectively alongside non-verbal communication, such as 3D models/artifacts produced iteratively in computer. Participants spent time developing the design concept, discussing design detail and referring to the design task. Design discussions advanced when every action of the designer produced visual information which initiated the next level of design action. This only can be done if the design communication media is able to provide such continuous visual feedback to the designer, thus maintaining a successful design interaction. Aligning with the concept of Kolko (2010), “interaction design is the creation of a dialogue between a person and a product,” the virtual instrument facilitates design dialogue through immersive interaction.

### Social Communication

The results of protocol analysis show that the design conversation extended beyond the task-related conversation. This indicates that the continuity of the conversation was naturally free-flowing. The design discourse focused on designing an urban form, with discussions extending beyond building shapes, size, space and types. Sometimes, participants talked about the impact of new design on the environment, on social cohesion and on inclusivity of the neighborhood. This proves that the dialogue exchange facilitated the transfer of subject-specific knowledge among the participants. Sometimes, through the inclusion of jokes, the designers came up with new design ideas, providing opportunities for social interaction with fellow design members.

### Communication Control and Communication Technology

The analysis of Communication Control indicates that there were design interruptions from the designers. This proves that the participant monitoring the design ideas on the 80-inch display screen understood the design ideas generated by the designers in the IVE. The Interruption due to Instrument indicates that the designers faced interruptions due to unfamiliarity with the IVE instruments. However, despite the interruption of the instruments, the result for Communication Technology shows that designers communicated meaningfully.

## Conclusion

This research demonstrates that the IVE-facilitated instrument has the capacity to allow laypeople to actively take part as designers in the early stage of an urban design process. The design set-up involving immersive and non-immersive VE, along with the framing of the design task, makes a case for further involving stakeholders in an urban design process. Urban design is a complex matrix which requires spontaneous participation of local knowledge and input to achieve an inclusive outcome. The instrument can empower non-experts to actively and collectively take part in the initial stage of design ideation and generation of an urban design process.

## Data Availability Statement

All data-sets generated for this study are included in the manuscript and can be requested to authors.

## Ethics Statement

The Victoria University of Wellington Human Ethics Committee has approved the research (#0000025705).

## Author Contributions

The authors confirmed being the sole contributors of this work and approved it for publication.

### Conflict of Interest

The authors declare that the research was conducted in the absence of any commercial or financial relationships that could be construed as a potential conflict of interest.
